# Human Metapneumovirus Inhibits IFN-β Signaling by Downregulating Jak1 and Tyk2 Cellular Levels

**DOI:** 10.1371/journal.pone.0024496

**Published:** 2011-09-19

**Authors:** Junping Ren, Deepthi Kolli, Tianshuang Liu, Renling Xu, Roberto P. Garofalo, Antonella Casola, Xiaoyong Bao

**Affiliations:** 1 Department of Pediatrics, University of Texas Medical Branch, Galveston, Galveston, Texas, United States of America; 2 Departments of Microbiology and Immunology, University of Texas Medical Branch, Galveston, Galveston, Texas, United States of America; 3 Sealy Center for Vaccine Development, University of Texas Medical Branch, Galveston, Galveston, Texas, United States of America; George Mason University, United States of America

## Abstract

Human metapneumovirus (hMPV), a leading cause of respiratory tract infections in infants, inhibits type I interferon (IFN) signaling by an unidentified mechanism. In this study, we showed that infection of airway epithelial cells with hMPV decreased cellular level of Janus tyrosine kinase (Jak1) and tyrosine kinase 2 (Tyk2), due to enhanced proteosomal degradation and reduced gene transcription. In addition, hMPV infection also reduced the surface expression of type I IFN receptor (IFNAR). These inhibitory mechanisms are different from the ones employed by respiratory syncytial virus (RSV), which does not affect Jak1, Tyk2 or IFNAR expression, but degrades downstream signal transducer and activator of transcription proteins 2 (STAT2), although both viruses are pneumoviruses belonging to the *Paramyxoviridae* family. Our study identifies a novel mechanism by which hMPV inhibits STAT1 and 2 activation, ultimately leading to viral evasion of host IFN responses.

## Introduction

Human metapneumovirus (hMPV) is a recently identified RNA virus belonging to the family of *Paramyxoviridae*, subfamily *Pneumovirinae*, genus *Metapneumovirus* [1]–[3] Since its discovery, hMPV has been recognized as a leading cause of lower respiratory infection in infants and children worldwide, second only to respiratory syncytial virus (RSV) [2], [4]–[6]. Virtually all children older than five years show serologic evidence of hMPV infection [3]. The virus is also associated with hospitalization and disease severity among elderly and immunocompromised patients with respiratory tract infections [7], [8].

Respiratory epithelial cells are the primary target of hMPV infection. An important feature of the host response to viral infections is the production of interferons (IFNs), a group of cytokines that activate an array of cellular genes that are critical in restricting viral replication and modulating adaptive immunity. Type I IFNs (IFN-α and IFN-β) are the key mediators produced by airway epithelial cells infected with paramyxoviruses [9]–[12], including hMPV [13]. Secreted IFN-α/β bind to IFN-α/β receptors (IFNAR) leading to dimerization of the two subunits, IFNAR1 and IFNAR2. IFNAR1 and IFNAR2 then undergo conformational changes resulting in the activation of the Janus tyrosine kinase (Jak)/signal transducer and activator of transcription protein (STAT) pathway. Tyrosine kinase 2 (Tyk2), a kinase belonging to the Jak family, is constitutively bound to IFNAR1 [14]. Tyk2 phosphorylates IFNAR1 at tyrosine residue 466 (Y466) and creates a docking site for STAT2 [15]. Subsequently, Tyk2 phosphorylates STAT2 at tyrosine 690 (Y690). Phosphorylation of STAT2 Y690 creates a new docking site for the SH2 domain of STAT1 [16], [17], which is subsequently phosphorylated at Y701 by IFNAR2 bound-Jak1 [18]. Phosphorylated STAT1 and STAT2 then dimerize and bind to IRF-9 [19]. This newly formed heterotrimer, IFN-stimulated gene factor 3 (ISGF3), translocates to the nucleus where it binds to IFN-stimulated response elements (ISRE) to initiate transcription of the interferon-stimulated genes (ISGs), leading to the establishment of an antiviral state within the host cell.

Almost all mammalian viruses, including paramyxoviruses, have developed strategies to avoid host immune responses by attacking the IFN system, which include direct targeting of signaling molecules belonging to the Jak/STAT signaling pathway, and increasing the expression or activity of endogenous cellular key regulators, such as suppressor of cytokine signaling (SOCS) proteins, protein tyrosine phosphatases (PTPs) and protein inhibitor of activated STATs (PIAS), to downregulate Jak/STAT-dependent signaling [20]–[23]. We have previously demonstrated that airway epithelial cells produce type I IFNs upon hMPV infection [13]. Recently, hMPV has been shown to antagonize IFN-α signaling through inhibition of STAT1 phosphorylation [24]. However, the mechanism leading to impaired STAT1 phosphorylation by hMPV infection was not identified. In this study, we describe a mechanism that contributes to hMPV antagonism of type I IFN signal transduction in human airway epithelial cells. We found that hMPV infection was associated with decreased cellular level of Jak1 and Tyk2, due to enhanced proteosomal degradation and reduced gene expression, as well as decreased surface expression of type I IFN receptor. Jak1 and Tyk2 degradation was viral-replication dependent, as infection with UV-inactivated hMPV failed to affect Jak1 and Tyk2 cellular levels. On the other hand, RSV, a close *Paramyxoviridae* family member, disrupts type I IFN-mediated signaling in airway epithelial cells through STAT2 degradation, without affecting the transcription and expression of Jak1 and Tyk2. Inhibition of Jak/STAT signaling by hMPV likely results in modulation of host cellular antiviral genes, allowing evasion of the airway defense response and the establishment of a productive infection. Given our limited knowledge of hMPV-induced pathophysiology of airway disease, a better understanding of the interplay between the virus and host immune defenses will help in designing new attenuated live vaccine candidates and in developing new antiviral treatment strategies.

## Results

### hMPV-induced STAT-dependent gene transcription requires Jak/STAT signaling

**Figure 1:**
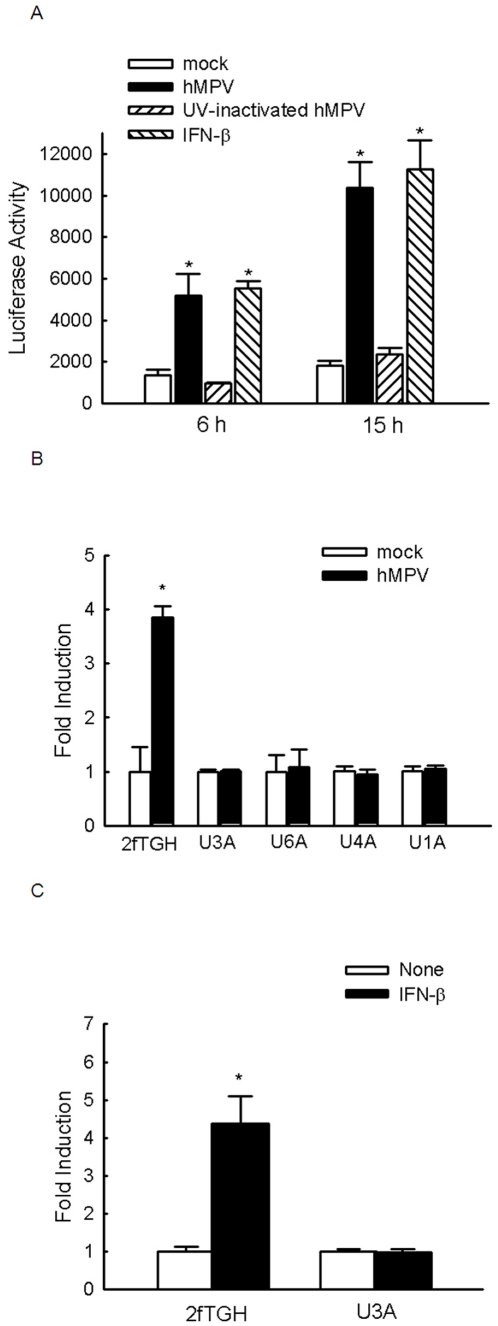
Induction of type I IFN signaling by hMPV infection. (**A**) A549 cells were transfected with a luciferase reporter plasmid containing five repeats of ISRE-binding sequence from ISG54 (pISRE-luc) for 30 h. Cells were then mock infected, or infected with live or UV-inactivated hMPV, at an MOI of 0.5, and harvested at various times p.i. to measure luciferase activity. IFN-β (1000 units/ml) treatment was used as a positive control. (**B**) Fibroblast cells 2fTGH and mutants lacking Jak1 (U4A), Tyk2 (U1A), STAT1 (U3A) or STAT2 (U6A) were transfected with pISRE-Luc for 30 h, followed by mock infection or infection with hMPV, at an MOI of 3, for 15 h. Cells were then lysed to measure luciferase activity. (**C**) pISRE-luc-transfected 2fTGH and U3A cells were either mock treated or treated with IFN-β for 15 h, followed by luciferase activity measurement. In all experiments, luciferase was normalized to β-galactosidase reporter activity. Data are representative of three independent experiments and are expressed as mean ± standard error (SE) of normalized luciferase activity. *, *P*<0.05, relative to mock-infected/treated cells.

Type I IFNs are a family of cytokines that mediate host resistance to virus infection by induction of ISGs via activated Jak/STAT signaling pathway. To confirm ISG transcription by hMPV-induced type I IFN, cells were transfected with a luciferase reporter plasmid containing five copies of the ISRE site derived from ISG54 gene promoter (pISRE-Luc), which binds transcription factors belonging to the STAT family, followed by hMPV infection. Cells were harvested at various times post-infection (p.i.) to measure luciferase activity. Mock infection and IFN-β treatment were used as negative and positive controls, respectively. We found that infection of A549 cells with hMPV, at an MOI of 0.5, caused a significant increase in the luciferase activity, compared to mock-treated cells, indicating that hMPV induces transcription of ISRE-dependent genes. hMPV replication was required for viral-induced ISRE-dependent gene transcription, as UV-inactivated hMPV failed to induce significant luciferase activity (Fig. 1A).

Jak1, Tyk2, STAT1, and STAT2 are essential signaling molecules for IFN-α/β-induced ISRE-dependent gene transcription [25]–[27]. To determine whether transactivation of pISRE-luc by hMPV occurs via the canonical Jak/STAT pathway, we investigated hMPV-induced ISRE-dependent gene transcription in Jak1, Tyk2, STAT1, or STAT2 deficient cells, in comparison to wild type cells. As shown in Fig. 1B, hMPV-infected 2fTGH cells had about 4 fold higher ISRE-dependent gene transcription at 15 h p.i. than uninfected cells. However, hMPV failed to induce luciferase activity in cells lacking STAT1, STAT2, Jak1, or Tyk2, suggesting that each component of the Jak/STAT pathway was necessary for hMPV-induced IFN-α/β signaling. IFN-β-treated 2fTGH cells showed a time-dependent increase in luciferase activity, which started at 6 h p.i., peaked at 15 and declined by 24 h p.i. compared to untreated cells (data not shown), while STAT1^−/−^ cells did not response to IFN-β treatment (Fig. 1C), confirming the response specificity of pISRE-Luc to IFN-β simulation, and the importance of STAT1 in regulating IFN-β-induced ISRE-dependent gene transcription.

### IFN-β-mediated signal transduction is inhibited by hMPV infection

**Figure 2:**
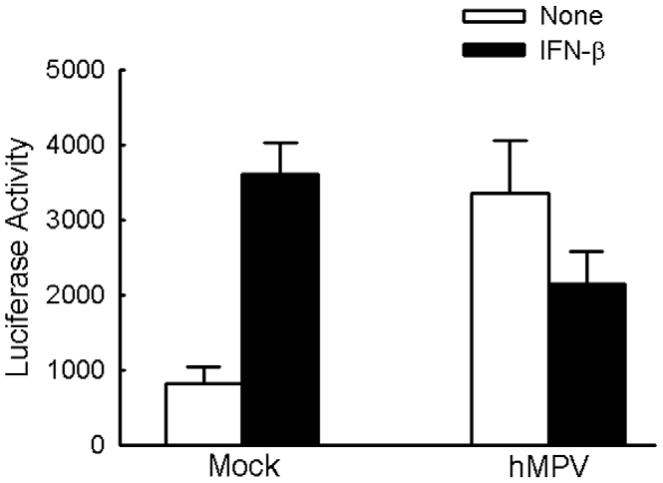
IFN-β-dependent signaling is inhibited by hMPV infection. A549 cells were mock- or hMPV-infected at an MOI of 0.5 for 24 h, and then transfected with pISRE-luc, followed by mock- or IFN-β treatment for an additional 15 h. For each plate luciferase was normalized to β-galactosidase reporter activity. Data are representative of two independent experiments and are expressed as mean ± SE of normalized luciferase activity.

Launching type I IFN signaling by virus infections is a critical step to initiate the expression of anti-viral ISGs. Simultaneously and progressively, viruses also develop strategies to counteract IFN responses to evade host defense [28]. For example, infection of RSV, a close family member to hMPV, increases baseline ISRE promoter activity, but simultaneously suppresses IFN-β responsiveness of the ISRE by degrading STAT2 [29]. Recently, it was reported that hMPV is also able to prevent IFN-α-mediated signaling and subsequent induction of ISGs[24]. Consistent with this observation, we also found that hMPV infection inhibited IFN-β-dependent ISRE transactivation, as cells treated with both hMPV and IFN-β did not have ISRE promoter activity equivalent to additive levels of ISRE promoter activity induced by respective treatment of hMPV and IFN-β (Fig. 2).

**Figure 3:**
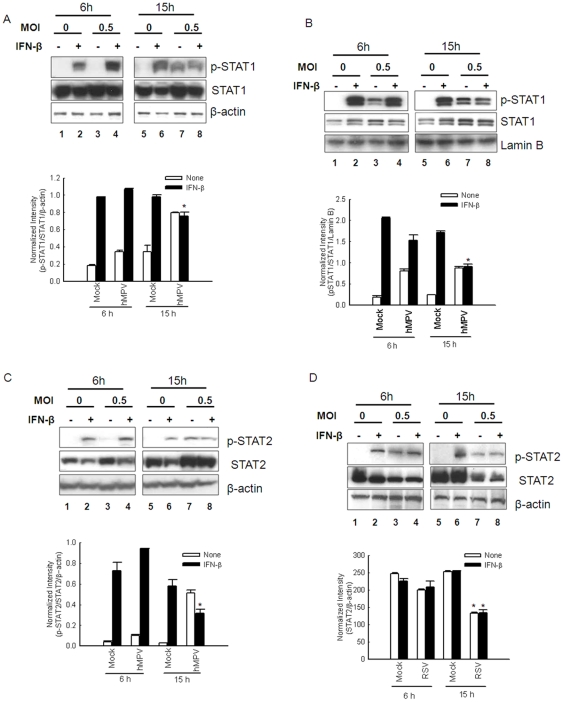
hMPV infection inhibits STAT1 and 2 phosphorylation. **A**) A549 cells were mock- or hMPV-infected at an MOI of 0.5, for 6 or 15 h, and then mock- or IFN-β-treatment for one additional hour. Total cells were harvested, followed by Western blot to determine the abundance of phosphorylated and non-phosphorylated STAT1. Membranes were stripped and reprobed for β-actin to document equal loading of the samples. Expression ratio of pSTAT1/STAT1 was normalized to β-actin. (**B**) A549 cells were infected with hMPV and/or treated with IFN-β as described in **A**. Nuclear fractions were prepared followed by Western blot using anti phospho-STAT1 (p-STAT1) and STAT1 antibodies. Membranes were stripped and reprobed for lamin B, as loading control. The nuclear translocated ratio of pSTAT1/STAT1 was normalized to lamin B. (**C**) Total cell lysate were prepared as described in **A**, followed by Western blot to determine the abundance of phosphorylated and non-phosphorylated STAT2. Membranes were stripped and reprobed for β-actin as a control for equal loading of the samples. Expression ratio of pSTAT2/STAT2 was normalized to β-actin. (**D**) A459 cell were mock- or RSV- infected, at an MOI of 0.5, for 6 or 15 h, and then mock- or IFN-β-treated for one additional hour. Total cell lysates were prepared, followed by Western blot to determine the abundance of phosphorylated and total STAT2. Membranes were stripped and reprobed for β-actin. STAT2 expression was normalized to β-actin. For (**A**), (**B**) and (**C**), *, *P*<0.05, relative to mock-infected and IFN-β-treated treated cells at 15 h. For (**D**), *, *P*<0.05, relative to mock-infected cells at 15 h p.i., according to their respective IFN-β treatment condition. The results are representative of two to three separate experiments. Densitometric analysis of Western blot band intensities was performed using VisionWorksLS image acquisition and analysis software from UVP (Upland, CA). Data are presented as mean ± SE of arbitrary units.

Several paramyxoviruses have been shown to inhibit type I IFN signaling by interfering with STAT phosphorylation and stability [30]–[32]. To determine the effect of hMPV infection on STAT1 and STAT2 activation in response to IFN-β treatment, A549 cells were mock-infected or infected with hMPV, at an MOI of 0.5, for 6 or 15 h, followed by IFN-β stimulation for 1 h, time of maximal STAT1 phosphorylation in response to the used concentration of IFN-β treatment (data not shown). Cells were then harvested to determine levels of STAT1 tyrosine phosphorylation. We found that STAT1 phosphorylation was significantly induced in A549 cells in response to both IFN-β treatment alone and to hMPV infection at 15 h p.i., with no changes in the amount of total cellular STAT1 (Fig. 3A). Additional IFN-β treatment failed to induce more STAT1 phosphorylation in cells infected with hMPV for 15 h, compared to mock-infected IFN-β-treated cells, indicating that hMPV infection inhibits type I IFN-induced STAT1 phosphorylation, similar to what has been reported for RSV [29].

Because some paramyxoviruses are able to prevent type I IFN–mediated signaling via by impairing STAT nuclear translocation translocation is a key process for STAT1 [33], [34], we investigated the possibility that this mechanism contributes to the inhibition of IFN-β-mediated signaling by hMPV. As shown in Fig. 3B, nuclear levels of both phosphorylated and regular STAT1 were significantly enhanced by IFN-β treatment or hMPV infection. At 6 h p.i., IFN-β-induced STAT1 phosphorylation in the nuclear compartment was not significantly affected by hMPV infection. However, at 15 h p.i., cells infected and stimulated with IFN-β had much less phosphorylated STAT1 than cells treated with IFN-β alone (Fig. 3B, upper panel, lane 8 vs. lane 7 vs. lane 6). Total nuclear STAT1 levels in response to IFN-β treatment were also affected by hMPV infection, as cells receiving both treatments did not exhibit an amount of STAT1 equivalent to additive levels induced by hMPV infection and IFN-β stimulation alone (middle panel: lane 4 vs lane 3+ lane 2, lane 8 vs lane 6+ lane 7). There was no cytoplasmic protein contamination of the nuclear extracts, as shown by the absence of β-tubulin in these samples (Fig. S1).

Western blot analysis was also used to determine whether hMPV inhibition of IFN-β response involved changes in STAT2 activation. As shown in the upper panel of Fig. 3C, phosphorylated STAT2 was undetectable in mock-infected A549 cells, but was markedly induced by IFN-β stimulation. In hMPV-infected cells, STAT2 phosphorylation was detectable at 15 h p.i. In this condition, IFN-β treatment of hMPV-infected cells did not result in a further increase of STAT2 phosphorylation (lane 8 vs lane 7 and lane 6), suggesting that hMPV infection also affects IFN-β-induced STAT2 activation. hMPV infection was associated with a moderate increase in STAT2 levels, compared to mock-infected cells, which is the opposite of what we observed in RSV-infected cells, where there was significant STAT2 degradation (Fig. 3D), consistent with what has been previously reported [29], [35].

### hMPV infection facilitates Jak1 and Tyk2 degradation

**Figure 4:**
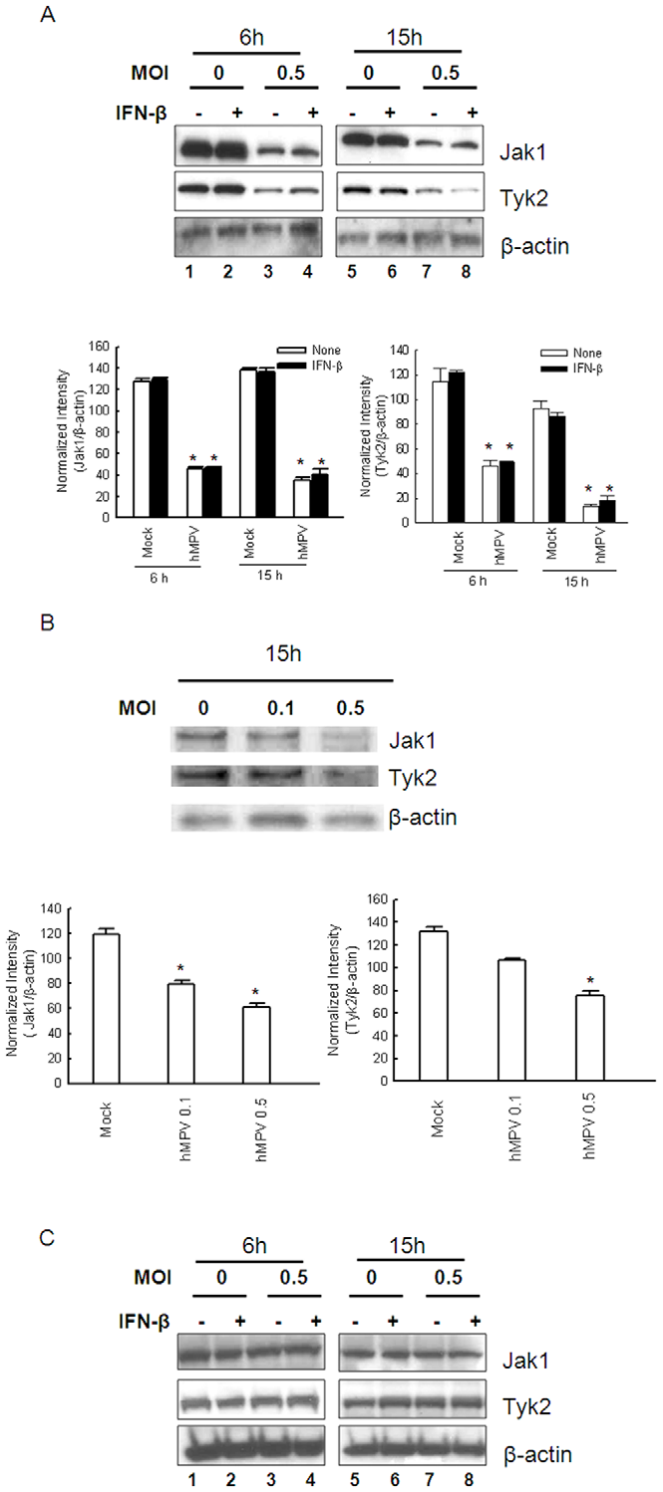
hMPV infection affects cellular levels of Jak1 and Tyk2. A549 cells (**A**) or Vero cells (**B**) were mock- or hMPV-infected, at an MOI of 0.5, for 6 or 15 h, followed by mock or IFN-β treatment for 1 h. Cells were harvested to prepare total cell lysates. Abundance of total Jak1 and Tyk2 was analyzed by Western blot, followed by densitometric analysis of Western blot band intensities using VisionWorksLS image acquisition and analysis software from UVP (Upland, CA). The band intensities were then normalized to β-actin levels. Data are representative of two to three independent experiments and are expressed as mean ± SE of normalized gene expression.*, *P*<0.05, relative to mock-infected cells, according to their respective IFN-β treatment condition and time point of p.i. (**C**) A549 cells were mock- or RSV-infected, at an MOI of 0.5, for 6 or 15 h, followed by mock or IFN-β treatment for 1 h. Cells were harvested to prepare total cell lysates. Abundance of total Jak1 and Tyk2 was analyzed as described in A and B. Data are representative of two to three independent experiments.

To determine whether the inhibitory effect of hMPV infection on IFN-β-induced STAT activation was the result of a disruption in the upstream cellular signaling pathway, we assessed protein abundance of Jak1 and Tyk2 in response to hMPV infection and/or IFN-β treatment. Western blot analysis revealed that Jak1 and Tyk2 cellular abundance was markedly reduced in response to hMPV infection itself, and in cells infected and treated with IFN-β, suggesting that inhibition of IFN-β-induced STAT1 and STAT2 phosphorylation results from hMPV-induced degradation of these two molecules (Fig. 4A). In Vero cells, which lack genes encoding type I IFNs, hMPV infection also induced Jak1 and Tyk2 degradation (Fig. 4B), indicating that this effect is directly induced by the virus and is not IFN-β-dependent.

In contrast, abundance of Jak1 and Tyk2 was not changed by RSV infection (Fig. 4C), indicating that RSV and hMPV use different mechanisms to impair type I IFN signaling.

### hMPV-induced Jak1 and Tyk2 degradation is proteasome-dependent

**Figure 5:**
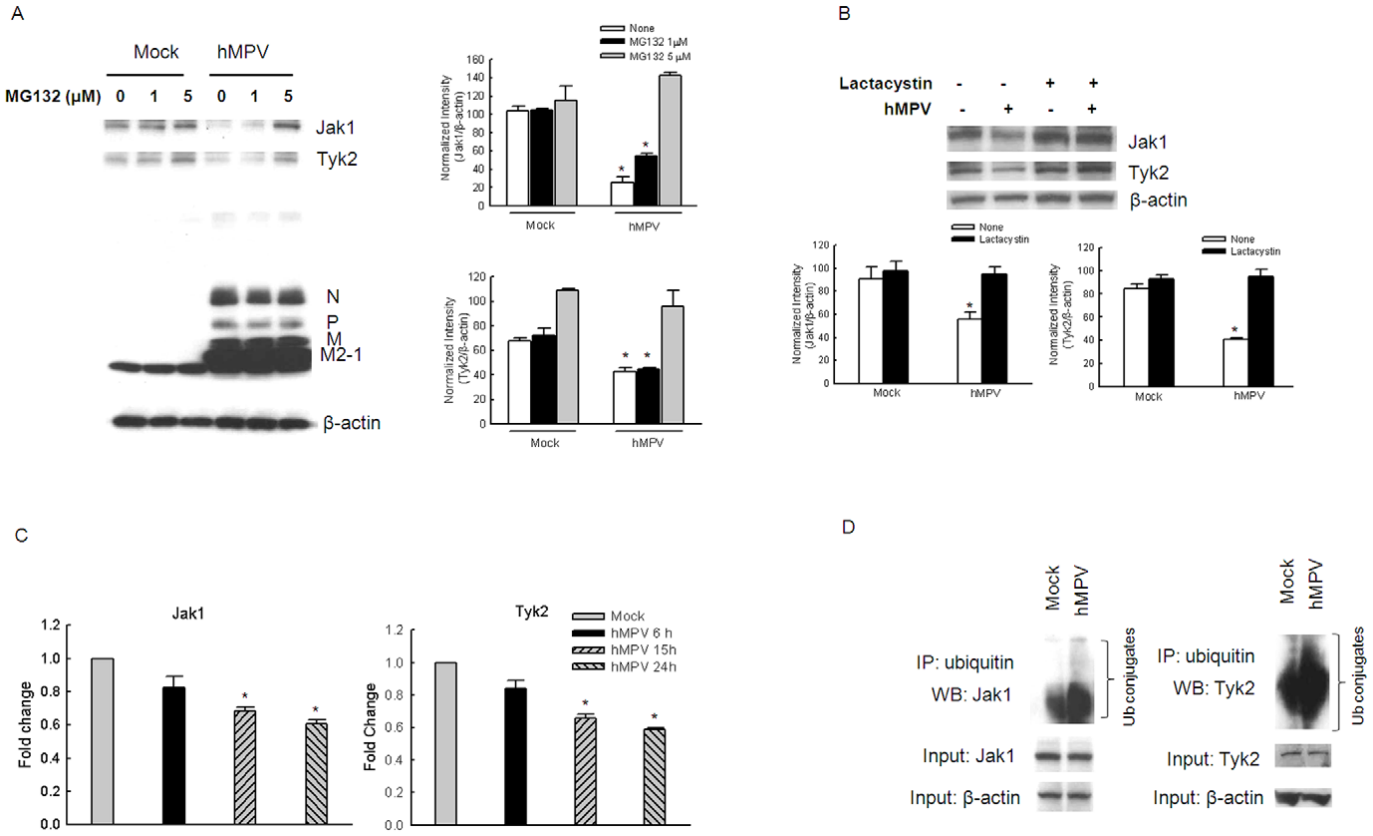
hMPV-induced Jak1 and Tyk2 degradation is proteasome-dependent. A549 cells were pretreated with 1 or 5 µM MG132 (A) or 10 µM lactacystin (**B**) for 1 h. Untreated cells were used as a control. After treatment, cells were mock-infected or infected with hMPV for 15 h and harvested to prepare total cell lysates. Jak1 and Tyk2 abundance was analyzed by Western blot. Membranes were also probed with anti-hMPV antibody to evaluate the effects of MG132 on viral replication. Membranes were stripped and reprobed with β-actin as a control for equal loading of the samples. *, *P*<0.05, relative to mock-infected cells, according to their respective MG132/lactacystin treatment conditions. (**C**) Total RNA was extracted from uninfected or infected A549 cells at 6, 15, and 24 h p.i., and used for Q-RT-PCR to determine changes in Jak1 and Tyk2 gene expression. 18S RNA was used as an internal control for normalization. Data are presented as fold changes of Jak1/Tyk2 mRNA levels in hMPV-infected cells compared to mock-infected. *, *P*<0.05, relative to mock-infected cells. (**D**) A549 cells were mock-infected or infected with hMPV in the presence of 5 µM MG132 for 6 h and harvested to prepare total cell lysate. Small aliquots were saved for Jak1 and Tyk2 abundance analysis by Western blot. Membranes were stripped and reprobed with β-actin as a control for equal loading of the samples. The rest of the samples were immunoprecipitated using an anti-polyubiquitin antibody, followed by Western blot using anti-Jak1 or anti-Tyk2 antibody. For all the experiments, data are representative of two to three independent experiments and are expressed as mean ± SE of normalized gene transcription or translation.

Proteasome-dependent degradation of Jak/STAT pathway components has been shown to be an important mechanism for paramyxovirus evasion of host responses. For example, human parainfluenza virus type 2 and RSV both induce downregulation of STAT2 levels via a proteasome-dependent pathway [36], [37]. To investigate this possibility, A549 cells were pretreated with the cell-permeable, nonspecific proteasome inhibitor MG-132 or the proteasome specific inhibitor lactacystin [38], [39], infected with hMPV, and harvested to measure Jak1 and Tyk2 levels in treated versus untreated cells by Western blot. We found that hMPV infection failed to reduce Jak1 and Tyk2 levels in A549 cells pretreated with 5 µM of MG132 or 10 µM of lactacystin (Fig. 5A and B). Because MG132 treatment has been previously shown to affect viral replication [40], [41], we examinated levels of hMPV protein synthesis in infected cells treated with MG132 by Western blot. There was no significant difference in hMPV protein expression in infected cells in the presence or absence of MG132 treatment, indicating that inhibition of Jak1 and Tyk2 degradation was not due to changes in viral replication (Fig. 5A).

In addition, hMPV infection also affected Jak1 and Tyk2 gene expression, as shown by a progressive decrease in Jak1 and Tyk2 mRNA levels in viral-infected cells (Fig. 5C), while there were no changes in Jak1 and Tyk2 expression in response to RSV infection (data not shown). On the other hand, STAT1, STAT2, IFNRA1, or IFNRA2 mRNA levels were unaffected by hMPV infection (data not shown).

Function of the ubiquitin-proteasome pathway is essential for many fundamental cellular processes, including the regulation of signaling pathways in the context of viral infections [42]. It has been previously shown that RSV degrades STAT2 by using the Elongin-Cullin E3 ligase, which is a ubiquitin ligase (E3) enzyme [35]. Therefore, we investigated whether hMPV degraded Jak-1 and Tyk-2 in an ubiquitin-proteasome-dependent pathway. A549 cells were mock infected or infected with hMPV for 6 h in the presence or absence of 5 µM of MG132. Total cell lysates were immunoprecipitated by an anti- polyubiquitin antibody (Santa Cruz, Biotechnology, Santa Cruz, CA), followed by Western blot using anti-Jak1 or anti-Tyk2 antibodies (Cell Signaling). We found that hMPV-infected cells had more ubiquitinated Jak1 and Tyk2 than mock-infected cells (Fig. 5D), suggesting the involvement of an ubiquitin-proteasome pathway in the degradation of Jak1 and Tyk2 in the context of hMPV infection.

### hMPV infection modulates the surface expression of IFNAR1 subunits

**Figure 6:**
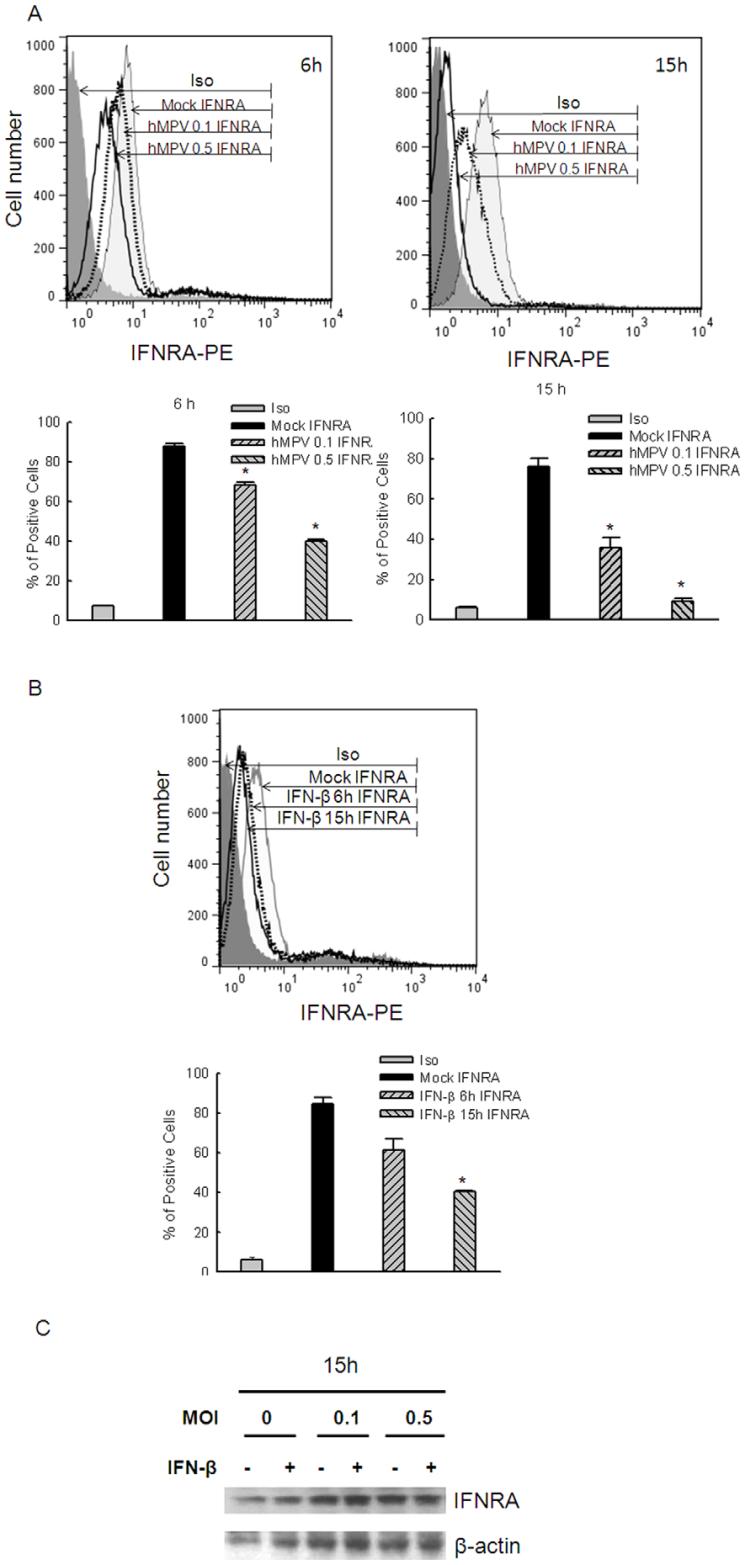
Surface expression of IFNAR1 subunits is reduced in hMPV-infected cells. (**A**) A549 cells were mock-infected or infected with hMPV at an MOI of 0.1 or 0.5, for 6 or 15 h. Cells were either stained with anti-IFNAR1 and isotype control antibody. After 1 h incubation at 37°C, cells were washed with PBS containing 5% FBS, followed by another incubation with anti-mouse FITC-conjugated secondary antibody at 37°C. (**B**) A549 cells were stimulated with 1,000 U/ml of IFN-β for various periods of time as indicated. Antibody staining was done as described in (A). Samples were analyzed on a FACSCAN flow cytometer equipped with BD FACSDiva software. Analysis was performed using FlowJo software (version 7.2.2; Tree Star Ashland, OR). % of IFNRA positive cells of mock- and hMPV-infected samples was compared. *, *P*<0.05, relative to mock-infected and IFNRA antibody-stained cells. Data are representative of two to three independent experiments, and are expressed as mean ± SE of IFNRA surface expression. (**C**) A549 cells were mock- or hMPV-infected, at an MOI of 0.1 or 0.5, for 15 h, and harvested to prepare total cell lysates. IFNAR1 abundance was determined by Western blot. Membranes were stripped and reprobed for β-actin as a control for equal loading of the samples. Data are representative of two to three independent experiments.

Since some viral infections have been shown to reduce cell surface expression of IFN receptors, as a mechanism to disrupt IFN-dependent signaling [43], [44], we investigated whether hMPV infection resulted in changes of IFNAR1 cell surface levels by using flow cytometry analysis of A549 and Vero cells. Compared to mock-infected cells, viral-infected A549 cells showed a time- and dose-dependent reduction of surface IFNAR1 expression (Fig. 6A). In contrast, surface expression of major histocompatibility complex-I (MHC-I) was either not changed at 6 h p.i., or considerably increased at 15 h p.i. (data not shown), suggesting that the decrease in IFNAR1 expression was unlikely due to altered cell viability. IFN-β treatment per se affected surface IFNAR1 expression in A549 cells (Fig. 6B), suggesting that hMPV-induced IFN-β secretion might contribute to reduced IFNAR1 surface levels. However, hMPV infection also reduced IFNAR1 surface expression in Vero cells, although to a less extent (data not shown), indicating that there is a ligand-independent pathway contributing to the suppression of IFNAR1 surface expression. Western blot analysis revealed that the amount of IFNAR1 in total cell lysate was comparable in mock- and hMPV-infected A549 cells (Fig. 6C), indicating that hMPV infection facilitated the internalization, but not degradation of IFNAR1.

### Impaired IFN-β response following hMPV infection is replication-dependent

**Figure 7:**
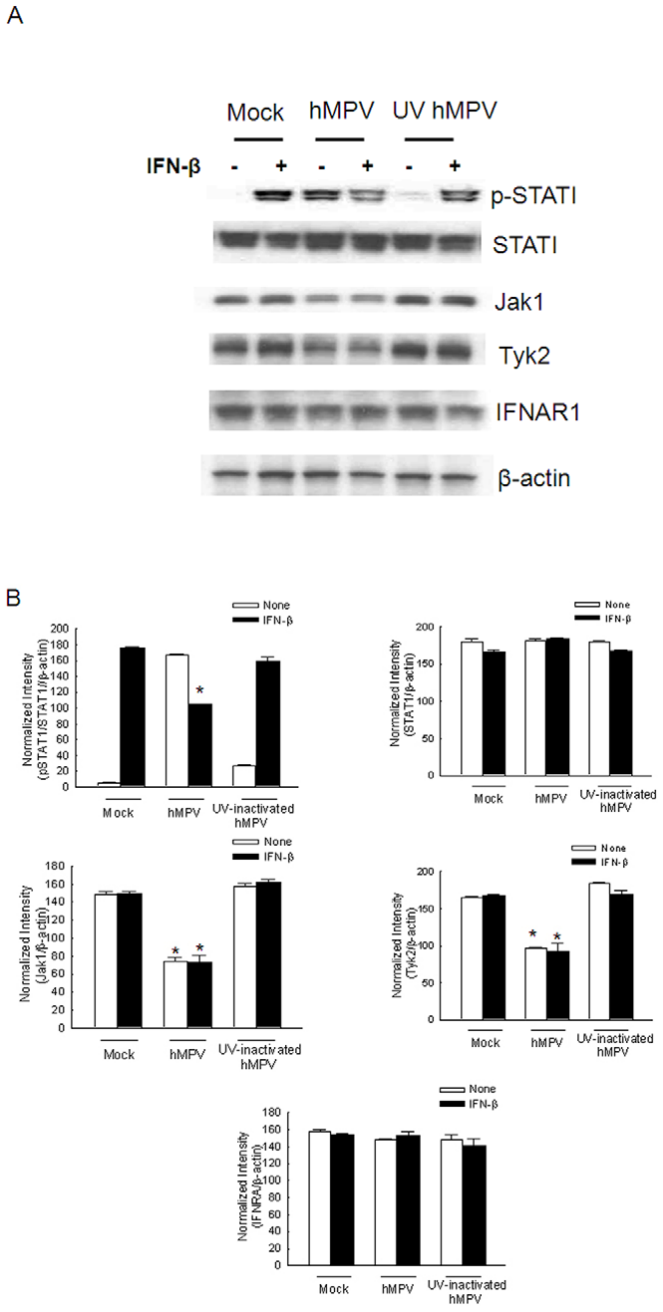
Inhibition of Jak/STAT1 signaling pathway is virus replication dependent. (**A**) A549 cells were mock-infected or infected with live hMPV or UV-inactivated hMPV for 15 h. Cells were then treated with medium or 500 U/ml of IFN-β for 1 h. Abundance of phosphorylated and total STAT1, Jak1 and Tyk2 in total cell lysates was then analyzed by Western blot. Membranes were stripped and reprobed with β-actin as a control for equal loading of the samples. (**B**) Densitometric analysis for **A**. densitometric analysis of band intensities was performed using VisionWorksLS Image Acquisition and Analysis Software from UVP (Upland, CA) according to the manufacturer's instruction. Data are representative of three independent experiments, and are expressed as mean ± SE of protein expression. *, *P*<0.05, relative to mock-infected cells, according to their respective IFN-β treatment conditions.

To examine the importance of viral replication on regulation of IFN-β-activated Jak/STAT pathway, STAT1 phosphorylation and the abundance of Jak1/Tyk2 were compared in A549 cells infected with live and UV-inactivated hMPV. Consistent with previous findings, phosphorylated STAT1 was undetectable in mock-infected A549 cells, but was readily detectable 1 h after IFN-β treatment. Infection with replicating, but not UV-inactivated virus, reduced IFN-β-induced STAT1 phosphorylation (Fig. 7). Similarly, infection with live, but not UV-inactivated virus, led to significant reduction of Jak1 and Tyk2 cellular levels (Fig. 7), indicating that inhibition of Jak/STAT pathway is dependent on viral replication and gene transcription, which is lost upon UV irradiation.

Taken together, these results indicate that hMPV modulates type I IFN-dependent Jak/STAT pathway by inducing proteasome-dependent degradation of Jak1 and Tyk2 and reducing surface expression of IFNAR1, ultimately leading to inhibition of STAT1 and STAT2 activation.

## Discussion

Epithelial cells are the primary target for respiratory virus infection. They actively participate in anti-viral responses by releasing and responding to type I IFNs. Previously, we showed that airway infection with hMPV resulted in the release of type I IFNs, IFN-α and IFN-β, both *in vitro* and *in vivo* [13], [45]. The secreted IFN-α/β likely binds to its receptors on cell surface, leading to the induction of gene transcription of anti-viral ISGs (Fig. 1A).

In the face of IFN-dependent host anti-viral defense strategies, many viruses simultaneously launch several counter mechanisms, which include inhibiting IFN synthesis, affecting type I IFN responses, and blocking expression of ISGs [46], [47]. We have recently shown that recombinant hMPV lacking glycoprotein G induces more type I IFN than its wild type counterpart, demonstrating the G protein of hMPV is inhibitory for viral-induced type I IFN synthesis [48]. In this study, we further demonstrated that hMPV infection also interferes with type I IFN signaling, leading to inhibition of IFN-β signaling transduction (Fig. 2). The inhibitory effect of hMPV on type I IFN signaling occurs at different levels of the signaling cascade, as the virus partially blocks Jak1 and Tyk2 gene transcription, facilitates Jak1 and Tyk2 degradation, and lowers IFNAR1 membrane expression, ultimately leading to inhibition of STAT1 and STAT2 activation. It has been shown that RSV, another important mucosal respiratory virus sharing similar clinical symptoms with hMPV, also inhibits type I IFN signaling in airway epithelial cells. However, the inhibition mechanisms employed by these two viruses are different. RSV seems only induce the downregulation of STAT2 without affecting Jak1 and Tyk2 (Fig. 4C). Unlike Jak1, which plays a significant role in mediating both type I and II IFN signaling, STAT2 only controls type I IFN signaling. Therefore, the selective degradation of STAT2 by RSV might account for its selective inhibition on type I IFN signaling in airway epithelial cells [49].

Other paramyxoviruses have also evolved a variety of strategies to counteract the antiviral effects of IFNs [50]. The V proteins of simian virus 5 (SV5) and human parainfluenza virus 2 (HPIV2) (genus *Rubulavirus*) are able to target STAT1 and STAT2 for polyubiquitination and proteasomal degradation [30], [31]. V proteins of Henipavirus and Nipah viruses, on the other hand, sequester STAT1 and STAT2 in high molecular mass cytoplasmic complexes without inducing their degradation. The C protein of Sendai virus disrupts IFN-signaling via inhibiting STAT1 and STAT2 tyrosine phosphorylation, while the V and C proteins of measles virus inhibit Jak1 phosphorylation and dissociate Jak proteins from the signaling complex [32]. Therefore, it is reasonable to hypothesize that hMPV encodes one or more proteins that are responsible for the inhibitory effects on type I IFN signaling. Currently, we are attempting to identify the viral components that are responsible for inhibition of type I IFN signaling.

In this study, we found that hMPV downregulates the expression of Jak1 and Tyk2 in infected airway epithelial cells as early as 6 h p.i. Infection with hMPV also reduces the expression of Jak1 and Tyk2 in Vero cells. Given the fact that Vero cells do not produce IFN-β, this result suggests that downregulation of Jak1 and Tyk2 by hMPV infection is probably independent of type I IFN. We also found that UV-inactivated hMPV is not able to inhibit the expression of Jak1 and Tyk2, suggesting that *de novo* viral gene expression and/or viral RNA replication is required for inhibition of expression of Jak1 and Tyk2. Although the global shutoff of host macromolecular synthesis plays an important role in pathogenesis of some viruses [51], our data suggest that this is not required for hMPV-mediated inhibition of Jak/STAT signaling, because reduced protein expression of Jak1 and Tyk2, and inhibition of STAT1 phosphorylation were not associated with an effect of hMPV on total STAT1 abundance. As hMPV-induced Jak1/Tyk2 degradation is proteasome-dependent, it is possible that a particular viral protein binds to specific host targets, facilitating the delivery of these targets to the proteasome, similar to what has been shown for RSV NS1 protein, that has the capacity to assemble ubiquitin ligase E3 enzymes to specifically target STAT2 for proteasome [35]. In addition to proteasome-dependent degradation, inhibition of mRNA transcription, by a yet to be identified mechanism, during the course of hMPV infection may also account for reduced expression of Jak1 and Tyk2.

In this study, we also detected reduced IFNAR1 surface expression in hMPV-infected airway epithelial cells, which occurred as early as 6 h p.i. Both reduced surface expression of IFNAR and downregulation of Jak1 and Tyk2 might contribute to reduced phosphorylation of STAT1 and STAT2, leading to inhibition of type I IFN responses. Both A549 and Vero cells had lower surface expression of IFNAR1 following hMPV infection, while the total cellular abundance of IFNAR1 was comparable in mock- and hMPV-infected cells (Fig. 6C). Tyk2-null cells have reduced surface levels of IFNAR1 protein, and as a consequence, binding of IFN-α is severely reduced. Conversely, when complexed to Tyk2, IFNAR1 is stabilized on the plasma membrane [52], [53]. Therefore, our results suggest that increased internalization of IFNAR1 following hMPV infection might result from the degradation of Tyk2, which is ligand-independent. This conclusion is also supported by the fact that RSV, which does not facilitate Tyk2 degradation, did not induce internalization of IFNAR1 in Vero cells (data not shown). Ligand-dependent pathway may also contribute to IFNAR1 internalization, as IFN-β treated A549 cells also had lower surface IFNAR1 expression than untreated cells (Fig. 6B), and hMPV- induced IFNAR1 internalization in Vero cells was not as significant as that in A549 cells (data not shown).

In summary, these studies demonstrate that hMPV antagonizes type I IFN responses through mechanisms involving the regulation of STAT1, STAT2, Jak1, Tyk2, and surface expression of IFNAR1. Whether inhibition of type I IFN responses represents an individual event or a series of sequential events following infection needs to be further investigated. In addition, whether or not the inhibition of type I IFN signaling by hMPV plays a role in pathogenesis and severity of infection also needs to be investigated, as inhibition of IFN signaling may affect development of host adaptive immunity, leaving the host susceptible to reinfection [54]. Therefore, a better understanding of how hMPV inhibits the type I IFN pathway, and its consequences with regard to the innate and adaptive immune responses, is crucial for improving therapeutic approaches and the development of better vaccines against hMPV infection.

## Materials and Methods

### Cell culture and viral infection

All media for cell culture was from GIBCO (Invitrogen, Carlsbad, CA) and supplemented with 100 IU/ml penicillin and 100 µg/ml streptomycin. A549, human alveolar type II-like epithelial cells (ATCC, Manassas, VA), were maintained in F12K medium containing 10% (v/v) FBS. Monkey kidney Vero and LLC-MK2 cells (ATCC, Manassas, VA) were maintained in MEM supplemented with 5% and 10% fetal bovine serum, respectively. The fibroblast cell line, 2fTGH, and mutants of 2fTGH lacking Jak1, Tyk2, STAT1 or STAT2 were generously provided by Dr. George R. Stark (Cleveland Clinic, Cleveland, Ohio) and cultured in DMEM supplemented with 1% sodium pyruvate and 1% hygromycin B.

### Virus preparation and viral infection

The Canadian isolate hMPV83 was propagated in LLC-MK2 cells at 37°C in the absence of serum and in the presence of 1 µg of trypsin/ml (Worthington, Lakewood, NJ). Viruses were purified by sucrose gradient centrifugation, as previously described [45], [48]. Viral titer was determined by immunostaining in LLC-MK2 cells, as previously described [48], [55]. Cell monolayers were infected with hMPV at a multiplicity of infection (MOI) of 0.5 in serum-free medium containing antibiotics and 1 µg trypsin/ml for all experiments, unless otherwise stated. Cells treated with infection media plus sucrose were defined as mock-infected.

### Reporter gene assays

Reporter gene assays were used to assess STAT-dependent gene transcription. A luciferase reporter plasmid (pISRE-Luc, Stratagene, La Jolla, CA) containing direct repeats of the interferon-stimulated response element (ISRE) found in the promoter of the 54-kDa interferon-stimulated gene 54 (ISG54) was transfected into log phase A549 cells or fibroblasts in triplicate using FuGene 6 (Roche, Basel, Switzerland), as previously described [56]–[59]. The next day, cells were infected with hMPV at an MOI of 0.5 for various times as indicated, or treated with IFN-β as positive controls. Plates containing uninfected cells served as negative controls. Cells were lysed and luciferase and β-galactosidase reporter activities were independently measured, as previously described [60]. All experiments were performed in duplicate or triplicate. In experiments investigating the role of hMPV in modulating the IFN-β response, cells were first infected with hMPV at an MOI of 0.1 for 24 h and then transfected with 2 µg of pISRE-Luc. Medium was removed 15 h after transfection and replaced with FBS-free medium containing IFN-β (1,000 IU/ml). After a 6 h incubation, luciferase levels from lysed cells were measured as previously described [60].

### Cellular and nuclear extracts

A549 cells were mock infected or infected with hMPV for 6 or 15 h, and then treated with 1000 units/ml of IFN-β for 1 h. Total cell lysates were then prepared by adding ice-cold lysis buffer (50 mM Tric-HCl, pH 7.4, 150 mM NaCl, 1 mM EGTA, 0.25% sodium deoxycholate, 1 mM Na_3_VO_4_, 1 mM NaF, 1% Triton X-100 and 1 µg/ml aprotinin, leupeptin and pepstatin). After incubation on ice for 10 min, the lysates were collected and detergent insoluble materials were removed by centrifugation at 4°C at 14,000 g. To investigate nuclear translocation of STATs, nuclear extracts from the cells were prepared using hypotonic/nonionic detergent lysis, according to the Schaffner protocol [61]. To prevent contamination with cytoplasmic proteins, isolated nuclei were purified by centrifugation through 1.7 M sucrose buffer A for 30 min, at 14,000 g before nuclear protein extraction, as previously described [62]. Cellular and nuclear extracts were then quantified using BCA Protein Assay Kit from Thermo Fisher Scientific (Thermo Fisher Scientific, Waltham, MA).

### Western blot analysis

Nuclear extracts or total cell lysates were fractionated by SDS-PAGE, and transferred to polyvinylidene difluoride membranes. Membranes were blocked with 5% milk, 0.5% Tween-20 in Tris buffered saline (TBS), and incubated with the proper primary antibodies according to the manufacturer's instruction. Primary antibodies for detection of p-Jak1 (Y1022/1023), Jak1, p-Tyk2 (Y1054/1055), Tyk2, p-STAT1 (Y701), STAT1, p-STAT2 (Y690), and STAT2 were from Cell Signaling (Cell Signaling Technology, Inc, Danvers, MA). Primary antibodies against IFNAR1 and anti-total ubiquitin were purchased from Santa Cruz (Santa Cruz Biotechnology, Santa Cruz, CA). Primary antibody against hMPV was a gift from Medimmune (Medimmune, Mountain View, CA). Appropriate horseradish peroxidase-coupled secondary antibody was then used and proteins were detected by enhanced chemiluminescence assay (Amersham, Piscataway, NJ). Equal loading of proteins was evaluated by stripping and reprobing the membranes with anti-β actin or anti-lamin B antibodies.

### Quantitative real-time PCR analysis

A549 cells were either mock infected or infected with hMPV at an MOI of 1. At the indicated time points, total RNA was isolated using Trizol (Invitrogen, Carlsbad, CA). Synthesis of cDNA was performed using 1 µg total RNA in a 20 µl reaction using the reagents in the Taqman Reverse Transcription Reagents Kit, Applied Biosystems, Inc. (ABI), Foster City, CA). Q-PCR amplifications (performed in triplicate) were done using 2 µl cDNA in a total volume of 25 µl containing SYBR Green PCR Master Mix (ABI, Foster City, CA) as specified by the manufacturer. The final concentration of the paired primers was 900 nM. Sequence information is available upon request. Real-time PCR was performed in an ABI 7500 thermocycler. Triplicate cycle threshold (*C_T_*) values were analyzed by the comparative ABI *C_T_* (ΔΔ*C_T_*) method. The relative amount of transcribed mRNA was normalized relative to the level of endogenous 18s RNA from each individual sample.

### Flow cytometry

To determine the relative IFNAR1 surface expression, confluent A549 cells or Vero cells were infected with hMPV or mock treated for 6 or 15 h. Cells were detached from the culture plate using Accutase™ enzyme cell detachment medium (eBioscience, San Diego, CA), and then washed twice with ice-cold wash solution (PBS with 5% heat-inactivated FCS). Cells were then incubated with mouse anti-IFNAR1 Abs (PBL, Piscataway, NJ) for 30 min. followed by staining using a FITC goat anti-mouse secondary antibody for another 30 min. Cells were then washed and fixed in 1% paraformaldehyde. In a separate set of experiments, A549 cells were stained with anti-MHC I antibodies to indirectly verify cell viability. Samples were analyzed using FACScan flow cytometer equipped with CellQuest software (both from Becton Dickinson Immunocytometry Systems, San Jose, CA). Analysis was performed in Flowjo software (Tree star, La Jolla, CA). Relevant isotype control antibodies were used for the mean intensity correction.

### Statistical Analysis

Statistical significance was analyzed using analysis of variance (ANOVA). *P* value of less than 0.05 was considered significant. Mean ± standard error (SE) is shown.

## Supporting Information

Figure S1 A549 cells were mock- or hMPV-infected at an MOI of 0.5, for 6 or 15 h, and then mock- or IFN-β-treated for one additional hour. Cells were harvested to prepare nuclear extract, which were probed by Western blot for-β-tubulin content, to exclude cytoplasmic protein contamination. A cytoplamic sample (cyto) from mock-infected cell was used as a positive control.
(TIF)
