# Supplementary figures and images for: Human Metapneumovirus Inhibits IFN-β Signaling by Downregulating Jak1 and Tyk2 Cellular Levels
Source: PLoS One. 2011 Sep 19;6(9):e24496. doi: 10.1371/journal.pone.0024496 (PMC3176284; doi:10.1371/journal.pone.0024496)

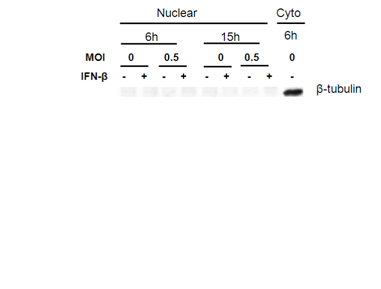

Supplement: Figure S1 — A549 cells were mock- or hMPV-infected at an MOI of 0.5, for 6 or 15 h, and then mock- or IFN-β-treated for one additional hour. Cells were harvested to prepare nuclear extract, which were probed by Western blot for-β-tubulin content, to exclude cytoplasmic protein contamination. A cytoplamic sample (cyto) from mock-infected cell was used as a positive control. (TIF) [file pone.0024496.s001.tif]
